# Book Reviews

**Published:** 1897-05

**Authors:** 


### Book Reviews

Twentieth Century Practice of Medicine. Vol. x. Diseases of the brain. William Wood & Co.

Unusual discrimination has been displayed in the classifica- on of brain diseases in this volume. Materials for the articles have been collected from current medical literature and the individual cases of the writers as well as from standard authors. The result shown is all that could be desired in a modern work. Hemorrhagic encephalitis takes its place among well described diseases. Special care has been bestowed upon the cellular diseases of the cord.

---

Inebriety. Its Source, Prevention and Cure. By Chas. F. Palmer.

This little work published by Fleming H. Revell Company is well written and neatly gotten up. The author takes the position of all advanced students of Inebriety and Drug Habits; viz., that certain individuals inherit a predisposition which develops in some cases Alcoholism, in others Neurasthenia, Chorea, or others Neurosis.

Taking this position he advocates the only humane and rational treatment; as preventive, careful development and raising of children inheriting neurotic tendencies, by proper attention to diet and exercise, as well as mental training, thus growing a healthy mind in a healthy body.

In developed inebriety, he recommends confinement in an institution for sufficient time to build up the physical and mental powers, and then such a course of living as recommended in the case of children to overcome the constitutional vice.

C. C. S.

---

Senn's Pathology and Surgical Treatment of Tumors, W. B. Saunders, Philadelphia.

This work from the pen of Prof. Nicolas Senn will be greatly appreciated by both surgeon and pathologist. Although Prof. Senn's views concerning the etiology of inflammation and of neoplasms are not accepted in their entirety by some pathologists they may be accepted as admirable guides by the clinician and the medical teacher. His statement of theory is clear and forcible, while his citation of many clinical cases from his wide experience adds to the practical value of the work. We take great pleasure in commending this work to both clinician and student.

GUIDES FOR A SELECTION OF CONSUMPTIVES AMENABLE TO HIGH ALTITUDE TREATMENT, A. E. Tussey, M. D. Blakiston Pub. $1.50.

In reading this short and interesting book one is continually forced to regret that the author has seen fit to cloth so much that is original and suggestive in a style so involved and confusing, a style which frequently makes it difficult fully to grasp his meaning. A flowery style such, as he largely uses, is apt to lack in that clearness and conciseness so necessary in a scientific work, and an author makes a great mistake who tries to adorn what he considers the truth in a dress of fanciful rhetoric, forgetting that truth is ever represented as naked and adorned only by her own loveliness and by the light which she diffuses.

A full analysis and criticism of the book in view of the space at disposal is impossible, though there is in it along with not a little to criticise very much to warmly commend, but I may be pardoned for pausing to say that in these days, when the etiological role of the qacillus tuberculosis has been not only bacteriologically, but clinically, so satisfactorily demonstrated, the author's pronounced scepticism as to its important relation to phthisis pulmonalis is scarcely in line with his claim to a strictly scientific attitude as to the disease.

The deductions made from his clinical experience as to the relation of vital capacity to total expansion and their bearing on prognosis are most suggestive and we trust he will carry them further and base them on a large series of cases fully reported as they promise to add a valuable means to those already at our disposal for estimating the chances of recovery in any given case. We have found in the book much calculated to be of value, not only to him whose work lies chiefly in this line but fully as much to the general practitioner. Were some of the author's warning laid to heart it would result in a distinct advance in the results obtainable by climatotherapy and the reviewer feels he can safely recommend its purchase and careful study feeling sure that his brother practitioners while not agreeing with all that is said will have no reason to regret the time spent in its perusal.

---

### PROGNOSIS OF INJURY AT THE ELBOW JOINT.

Be very guarded of prognosis in cases of injury at the elbow A fracture into this joint, treated with the most far seeing precautions, may be followed by more or less stiffness and disability. Begin passive motion as early as possible, delaying only long enough to allow the first pain and reaction from the injury to subside; in most cases this will allow some manipulation of the joint by the end of the first week.—*International Journal of Surgery.*

